# Mounting Crowns and Bridge

**Published:** 1901-04-15

**Authors:** H. J. Goslee


Mounting Crowns and Bridge.—Were it possible to successfully manipulate gutta-percha, the advantages of ease of removal, insolubility, and the cushion-like effect afforded would render it practically ideal; but, unfortunately, it is difficult to successfully manipulate in extensive cases, and requires considerable time and much effort in single crowns.

I usually give preference to cement, but always observe the precaution of coating the interior of crown and post with a thin film of chloro-percha or gum shellac to facilitate removal in case of necessity.—*Dr. H. J. Goslee.*

